# Epigenetic Influences on Associations between Air Pollutants and Lung Function in Elderly Men: The Normative Aging Study

**DOI:** 10.1289/ehp.1206458

**Published:** 2014-03-06

**Authors:** Johanna Lepeule, Marie-Abele Catherine Bind, Andrea A. Baccarelli, Petros Koutrakis, Letizia Tarantini, Augusto Litonjua, David Sparrow, Pantel Vokonas, Joel D. Schwartz

**Affiliations:** 1 Exposure, Epidemiology, and Risk Program, Department of Environmental Health, Harvard School of Public Health, Boston, Massachusetts, USA; 2 Center of Molecular and Genetic Epidemiology, Department of Environmental and Occupational Health, Ca’ Granda Ospedale Maggiore Policlinico IRCCS Foundation, Università degli Studi di Milano, Milan, Italy; 3 Channing Laboratory, and; 4 Division of Pulmonary and Critical Care Medicine, Brigham and Women’s Hospital, Harvard Medical School, Boston, Massachusetts, USA; 5 VA Normative Aging Study, Veterans Affairs Boston Healthcare System and the Department of Medicine, Boston University School of Medicine, Boston, Massachusetts, USA

## Abstract

Background: Few studies have been performed on pulmonary effects of air pollution in the elderly—a vulnerable population with low reserve capacity—and mechanisms and susceptibility factors for potential effects are unclear.

Objectives: We evaluated the lag structure of air pollutant associations with lung function and potential effect modification by DNA methylation (< or ≥ median) at 26 individual CpG sites in nine candidate genes in a well-characterized cohort of elderly men.

Methods: We measured forced vital capacity (FVC), forced expiratory volume in 1 sec (FEV_1_), and blood DNA methylation one to four times between 1999 and 2009 in 776 men from the Normative Aging Study. Air pollution was measured at fixed monitors 4 hr to 28 days before lung function tests. We used linear mixed-effects models to estimate the main effects of air pollutants and effect modification by DNA methylation.

Results: An interquartile range (IQR) increase in subchronic exposure (3 to 28 days cumulated), but not in acute exposure (during the previous 4 hr, or the current or previous day), to black carbon, total and nontraffic particles with aerodynamic diameter ≤ 2.5 μm (PM_2.5_), carbon monoxide, and nitrogen dioxide was associated with a 1–5% decrease in FVC and FEV_1_ (*p* < 0.05). Slope estimates were greater for FVC than FEV_1_, and increased with cumulative exposure. The estimates slopes for air pollutants (28 days cumulated) were higher in participants with low (< median) methylation in *TLR2* at position 2 and position 5 and high (≥ median) methylation in *GCR*.

Conclusions: Subchronic exposure to traffic-related pollutants was associated with significantly reduced lung function in the elderly; nontraffic pollutants (particles, ozone) had weaker associations. Epigenetic mechanisms related to inflammation and immunity may influence these associations.

Citation: Lepeule J, Bind MAC, Baccarelli AA, Koutrakis P, Tarantini L, Litonjua A, Sparrow D, Vokonas P, Schwartz JD. 2014. Epigenetic influences on associations between air pollutants and lung function in elderly men: the Normative Aging Study. Environ Health Perspect 122:566–572; http://dx.doi.org/10.1289/ehp.1206458

## Introduction

By 2030, there will be 72.1 million people ≥ 65 years of age, representing 19% of the U.S. population, according to the Department of Health and Human Services (U.S. Department of Health and Human Services 2014). The aging process reduces physiological capacity, which makes the elderly more susceptible to many health threats. There is compelling evidence that short- and long-term exposure to ambient air pollution, especially due to traffic, adversely affect lung function (Brunekreef et al. 1995). However, most of the studies have focused on children. Although research examining the effects of air pollution on forced vital capacity (FVC) and forced expiratory volume in 1 sec (FEV_1_) in the elderly is warranted (Sandstrom et al. 2003), to our knowledge, these associations have not yet been investigated.

The underlying mechanisms linking air pollution and lung function are not fully characterized. Lung function has been shown to be strongly heritable, part of which is controlled by inflammatory genes (Sunyer et al. 2008). Consistent with this observation, many studies have reported associations between lung function and polymorphisms within genes coding for inflammatory, oxidative stress and innate immunity mediators, such as *CRP* (Sunyer et al. 2008), *IL-6* (He et al. 2009), *iNOS* (Islam et al. 2009), or *TLRs* (Budulac et al. 2012). Other studies have reported stronger associations between air pollution and pulmonary outcomes in subjects with such polymorphisms (Kerkhof et al. 2010; Yang et al. 2008). There is increasing evidence that epigenetic mechanisms may interact with genetic variation to influence disease pathogenesis and the inheritance of disease traits.

Methylation in CpG-rich regions within gene promoters is commonly associated with repressed gene expression, because it may impede the binding of transcription factors (Hashimshony et al. 2003). Studies in humans have reported associations between blood DNA methylation and cardiovascular diseases (Stenvinkel et al. 2007), respiratory health (Lepeule et al. 2012), and survival (Baccarelli et al. 2010).

We hypothesized that short-term exposure to traffic-related air pollutants would be associated with lung function decrease in a cohort of elderly men. Subsequently, we examined whether methylation level within or near the promoter region of selected genes related to inflammation, immunity, endothelial function, and oxidative stress may be a susceptibility factor for lung function impairment.

## Methods

*Study population and pulmonary health*. Our study included 776 elderly men living in the Boston, Massachusetts, area, enrolled in the Normative Aging Study cohort (Bell et al. 1966). Participants provided written informed consent, and the study was approved by the institutional review boards of all participating institutions. Subjects completed one to four clinical examinations between 1999 and 2009. Each visit took place in the morning after an overnight fast and smoking abstinence. At each visit, information about medication use (corticosteroids, sympathomimetic α and β, anticholinergics), pulmonary disorders, and smoking history were collected using the American Thoracic Society questionnaire (Ferris 1978).

Spirometric tests were performed following a strict protocol in accordance with American Thoracic Society guidelines, as previously reported (Sparrow et al. 1987). Spirometry was assessed in the standing position with a noseclip using a 10-L water-filled survey-recording spirometer and an Eagle II minicomputer (Warren E. Collins, Braintree, MA, USA). Values were adjusted by body temperature and pressure. A minimum of three acceptable spirograms was obtained, of which at least two were reproducible within 5% for both FVC and FEV_1_. Each technician underwent training before taking measurements for this study.

Methacholine challenge tests were conducted between 1984 and 2000 using procedures adapted from Chatham et al. (1982). We used data from the most recent test available for each subject at that visit. Participants with ischemic heart disease or baseline FEV_1_ < 60% of the predicted value were excluded, and some elected not to participate. Methacholine inhalations were administered at incremental doses corresponding to 0, 0.330, 1.98, 8.58, 16.8, and 49.8 μmol. Participants whose FEV_1_ declined by 20% in response to any of the doses at or before 8.58 μmol were classified as having airway hyperresponsiveness. Participants whose FEV_1_ did not decline by 20% in response to any of the administered doses, and participants who demonstrated a 20% decline in FEV_1_ at higher methacholine dosages (16.8 or 49.8 μmol) only were categorized as having no airway hyperresponsiveness.

*DNA methylation*. DNA was extracted from stored frozen buffy coat of 7 mL whole blood, using QiAmp DNA blood kits (QIAGEN; http://www.qiagen.com). DNA methylation was quantitated using bisulfite-polymerase chain reaction and pyrosequencing (Tost and Gut 2007) within or nearby the promoter regions of a total of nine genes: Carnitine O-acetyltransferase (*CRAT*), coagulation factor-3 (*F3*), glucocorticoid receptor (*GCR*), intercellular adhesion molecule (*ICAM1*), interferon-gamma (*IFN-*γ), interleukin-6 (*IL6*), inducible nitric oxide synthase (*iNOS*), 8-oxoguanine DNA glycosylase 1 (*OGG1*), and toll-like receptor 2 (*TLR2*). Primers and conditions were previously described (Lepeule et al. 2012). These genes were selected because they are expressed in leukocytes (AceView 2013) and are related to cardiorespiratory health, inflammation, and oxidative stress mechanisms (Lepeule et al. 2012; Poole et al. 2011; Sin and Man 2008). The promoter regions were located using Genomatix Software (Genomatix Software Inc., Ann Arbor, MI, USA) (see Supplemental Material, Table S1). For each gene, we measured 1–5 CpG sites (positions) located within or outside of CpG islands. DNA methylation analysis was repeated on each sample, and results were averaged to reduce assay variability. We used non-CpG cytosine residues as built-in controls to verify bisulfite conversion. The degree of methylation was expressed for each DNA locus as percent methylated cytosines over the sum of methylated and unmethylated cytosines. Because of assay failure and limited amounts of DNA available from each subject, DNA methylation analysis was successful on varying numbers of subjects for each sequence. All samples were analyzed consecutively by one laboratory technician.

*Environmental measurements*. Exposure to urban background pollution was estimated using fixed monitoring stations. Ambient concentrations of black carbon (BC) and of particles with aerodynamic diameter ≤ 2.5 μm (PM_2.5_) were measured hourly at Harvard Supersite, positioned at the top of a building located < 1 km from the medical center, where the participants’ visits took place. BC concentrations were measured using an aethalometer (Magee Scientific Inc., Berkeley, CA, USA), and PM_2.5_ mass concentrations were measured using a Tapered Element Oscillating Microbalance (TEOM model 1400A; Rupprecht and Patashnick Co., East Greenbush, NY, USA). TEOM measurements were corrected for loss of semivolatile particles during sampling, using a collocated gravimetric sampler. About 10% of hourly missing measurements for BC and PM_2.5_ were imputed through a linear regression, where each pollutant was regressed against season, long-term time trend, day of the week, mean temperature, relative humidity, barometric pressure, extinction coefficient, and previous and following day measurements of the pollutant (Zanobetti and Schwartz 2007). We regressed hourly PM_2.5_ concentrations against BC (mainly contributed by traffic sources in Boston) and used the residuals as a surrogate measure of nontraffic PM_2.5_ (Schwartz et al. 2005). Hourly carbon monoxide (CO), ozone (O_3_), and nitrogen dioxide (NO_2_) concentrations were measured by local state monitors (four monitors for CO and O_3_, five for NO_2_) and averaged over all monitors. The median distance of the participant homes was 20.8 km to the BC and PM_2.5_ monitoring site, and 20.2, 22.3, and 21.4 km, respectively, for the CO, O_3_, and NO_2_ monitors. We obtained temperature and relative humidity data from the National Weather Service Station at Logan Airport (Boston, MA, USA), located aproximately 12 km from the examination site.

Daily averages were calculated when at least 75% of the hourly values for a given day were available. For each pollutant concentration, we considered a range of short-term and subchronic exposure windows preceding each subject’s examination, including: 4 hr, 24 hr (lag 0), previous day (lag 1), and 3, 7, 14, and 28-day moving averages. Because all visits were scheduled for the morning, exposure windows were calculated from 0800 hours the day of the visit.

*Statistical analysis*. We studied the effects of air pollutants on lung function using a mixed linear model. Separate models were run for each pollutant, exposure window, and lung function measurement:

*Y_it_* = β_0_ + *u_i_* + β_1_ Air pollutant*_it_* + β_2_ X_2_*_it_* + … + β*_p_*X*_pit_* + ε*_it_*, [1]

where *Y_it_* was the log-transformed lung function measurement for participant *i* at visit *t*, β_0_ was the intercept, *u_i_* was the random effect, β_1_ was the effect of the air pollutant on lung function measurement, *X*_2_*_it_* to *X_pit_* were the *p*–1 covariates, and ε_it_ was the within-participant error. We selected the following adjustment covariates *a priori* and added a quadratic term whenever it was significant: age (linear and quadratic), ln(height) (linear and quadratic) and standardized weight (linear), race, education level, smoking status, cumulative smoking in pack-years, season of the medical examination (using sine and cosine of time), day of the week, visit number, temperature and relative humidity (matched on air pollutant exposure window), physician-diagnosed chronic lung conditions (asthma, emphysema, chronic bronchitis), methacholine responsiveness, medication use. Because participants with chronic lung conditions are expected to be sicker than average, we explored potential modification of air pollution effects (28-day moving average) by emphysema, chronic bronchitis, methacholine responsiveness (as an objective indicator of asthma), and chronic obstructive pulmonary disease [COPD; defined as GOLD (Global Initiative for Chronic Obstructive Lung Disease) stage II (FEV_1_/FVC < 70% and FEV_1_ < 80% predicted) or higher].

Several sensitivity analyses were performed. First, we adjusted models for cardiovascular diseases (coronary heart diseases, stroke), diabetes, and hypertension. Next, we excluded participants with physician-diagnosed asthma, emphysema, or chronic bronchitis, and participants with methacholine responsiveness. Finally, to adjust for the fact that healthier men are more likely to come back for subsequent visits, we applied inverse probability weighting (Hernan et al. 2006) using logistic regression to calculate the probability of having a subsequent visit given age, education level, body mass index, smoking status, pack-years, hypertension, cholesterol, diabetes, FEV_1_, asthma, emphysema, chronic bronchitis, methacholine responsiveness, and air pollutant concentration at previous visit. When estimating interactions between DNA methylation and pollutants, we repeated analyses excluding subjects with chronic lung conditions or taking lung-related drugs (corticosteroids, sympathomimetic α and β, anticholinergics).

We then investigated whether DNA methylation in selected genes influences susceptibility by modeling interactions between 28-day moving average air pollutant concentrations and methylation at individual CpG sites (26 total sites over 9 genes), with methylation dichotomized as high or low at each site based on the median of the distribution (Table 1). In addition, we estimated interactions with high or low methylation based on the average over all CpG sites within each gene. Because methylation of long interspersed nucleotide elements (LINE-1) and Alu repetitive elements has been associated with lung function (Lange et al. 2012), we also modeled interaction of LINE-1 and Alu methylation (dichotomized as high or low based on the median of the distribution) with air pollutant concentrations as a secondary analysis. LINE-1 and Alu methylation each were measured in three replicates and averaged for statistical analysis.

**Table 1:** Blood DNA methylation levels (percentage of 5-methylcytosine) in 1,515 visits, the Normative Aging Study, 1999–2009.

Gene and position	*n*	DNA methylation (mean ± SD)	Percentile		
			5th	50th	95th
*CRAT*	1,411
Position 1		1.7 ± 0.6	1.0	1.7	2.6
Position 2		4.7 ± 1.6	2.4	4.6	7.7
Mean		3.2 ± 1.0	1.7	3.2	5.1
*F3*	1,273
Position 1		1.5 ± 1.8	0.0	1.2	4.2
Position 2		1.6 ± 1.9	0.0	1.2	4.6
Position 3		3.0 ± 2.2	0.0	2.5	6.9
Position 4		1.4 ± 1.6	0.0	1.1	3.9
Position 5		4.5 ± 2.6	0.0	4.2	8.5
Mean		2.4 ± 1.3	0.9	2.2	4.5
*GCR*	1,283
Position 1		47 ± 6	36	47	55
*ICAM*	1,173
Position 1		5.8 ± 2.3	2.9	5.5	10.3
Position 2		3.5 ± 2.2	1.7	3.0	9.2
Position 3		3.8 ± 2.0	1.7	3.3	7.3
Mean		4.4 ± 1.8	2.3	4.0	8.1
*IFN-*γ	1,460
Position 1		82 ± 6	72	84	90
Position 2		87 ± 5	80	88	93
Mean		85 ± 5	76	86	91
*IL6*	1,469
Position 1		47 ± 12	27	47	66
Position 2		40 ± 11	22	40	58
Mean		43 ± 10	26	44	62
*iNOS*	1,017
Position 1		53 ± 8	39	53	66
Position 2		83 ± 9	65	85	97
Mean		68 ± 7	55	69	79
*OGG1*	915
Position 1		2.1 ± 2.6	0.0	1.2	7.4
Position 2		2.9 ± 2.6	0.0	2.3	8.3
Position 3		2.2 ± 2.0	0.0	1.9	6.1
Position 4		2.0 ± 2.2	0.0	1.5	6.5
Mean		2.3 ± 1.4	0.7	1.9	4.9
*TLR2*	1,172
Position 1		2.8 ± 1.8	0.0	2.5	6.1
Position 2		3.5 ± 2.0	0.0	3.3	7.1
Position 3		2.9 ± 2.0	0.0	2.6	6.3
Position 4		3.7 ± 2.1	0.0	3.4	7.3
Position 5		2.0 ± 1.9	0.0	1.8	5.6
Mean		3.0 ± 1.3	1.2	2.7	5.4

Air pollution, DNA methylation, and lung function were all time-varying variables. Models were also adjusted for the percentage of neutrophils, lymphocytes, basophils, eosinophils, and monocytes measured at each visit. Possible mediation of the effects of air pollution on lung function through DNA methylation was tested separately by including DNA methylation variables in the model for the main effects (Equation 1).

*p*-Values < 0.05 were considered statistically significant. All statistical analyses were conducted with SAS version 9.2 (SAS Institute Inc., Cary, NC, USA).

## Results

*Descriptive results*. Participants were mainly white, well educated, and former smokers (Table 2). At first visit, 88% of them were ≥ 65 years old. Spearman correlation between FVC and FEV_1_ was 0.90. Air pollutant concentrations were relatively low in terms of particles, with an average of 0.9 ± 0.4 μg/m^3^ for BC and 11 ± 7 μg/m^3^ for total PM_2.5_ (Table 3). BC concentrations explained 34% of the PM_2.5_ variability, suggesting that the remaining part was explained by nontraffic sources. The correlation between PM_2.5_ residuals and CO was low (*r*_S_ = 0.12). Because CO is a marker of traffic pollution, this low correlation confirms that BC accounts for most of the variability due to traffic PM_2.5_, and that the PM_2.5_ residuals are a marker of nontraffic particles.

**Table 2:** Characteristics of 776 men, the Normative Aging Study, 1999–2009.

Characteristic	Value
Participant characteristics at 1st visit	
Age (years)	72.3 ± 6.8
Race
Black	14 (1.8)
White	754 (97.2)
Missing	8 (1.0)
Height (cm)	173.5 ± 7.0
Weight (kg)	85.3 ± 14.3
Education (years)
< 12	30 (3.9)
12	187 (24.1)
13–15	219 (28.2)
> 15	333 (42.9)
Missing	7 (0.9)
Smoking status
Never	220 (28.3)
Current	33 (4.3)
Former	523 (67.4)
Pack-years^*a*^	21.6 ± 26.8
Asthma	46 (5.9)
Chronic bronchitis	53 (6.8)
Emphysema	29 (3.7)
Methacholine responsiveness	74 (9.5)
Missing	125 (16.1)
Corticosteroids	53 (6.8)
Sympathomimetic (α, β)	56 (7.2)
Anticholinergic	14 (1.8)
FVC (L)	3.3 ± 0.7
FEV_1_ (L 1st sec)	2.5 ± 0.6
Visit characteristics (*n *= 1,515)	
Season
Spring (March–May)	350 (23.1)
Summer (June–August)	430 (28.4)
Fall (September–November)	490 (32.3)
Winter (December–February)	245 (16.2)
Day of the week
Tuesday	71 (4.7)
Wednesday	394 (26.0)
Thursday	824 (54.4)
Friday	226 (14.9)
Visit number
1	776 (51.2)
2	501 (33.1)
3	192 (12.7)
4	46 (3.0)
Values are mean ± SD or *n* (%). ^***a***^Among current or former smokers.	

**Table 3:** Environmental characteristics 24 hr before lung function assessment for 1,515 visits, the Normative Aging Study, 1999–2009.

Characteristic	Mean ± SD	5th, 95th Percentiles	IQR	Spearman correlation coefficient				
				BC	CO	NO_2_	O_3_	PM_2.5_
Air pollutant (μg/m^3^)^*a*^
BC	0.9 ± 0.4	0.5, 1.1	0.6
CO	502 ± 285	299, 660	362	0.42
NO_2_	38 ± 12	30, 45	15	0.59	0.62
O_3_	47 ± 24	28, 60	33	–0.21	–0.29	–0.31
PM_2.5_	11 ± 7	6, 13	7	0.70	0.34	0.52	0.04
PM_2.5_ nontraffic^*b*^	0 ± 5	–3, 1	5	0.18	0.12	0.25	0.24	0.80
Weather
Temperature (ºC)	13 ± 9	7, 20	13
Relative humidity (%)	68 ± 16	56, 81	25
All *p*-values were < 0.05. ^***a***^Sample sizes were between 1,499 and 1,515, indicating very few missing data. ^***b***^Because nontraffic PM_2.5_ values are the residuals of the regression of PM_2.5_ against BC, the average was 0.								

**Figure 1:**
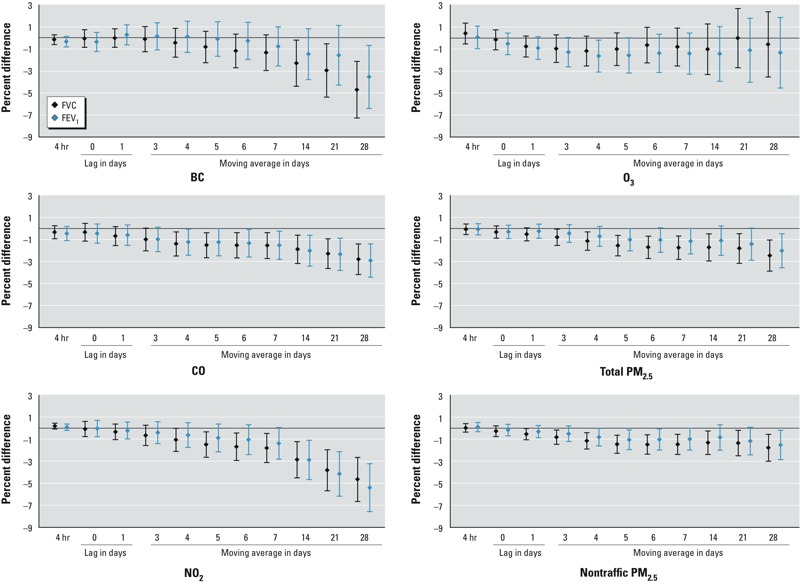
Percent difference in FVC and FEV_1_ (and 95% CIs) associated with 1 IQR increase in air pollutant concentration, the Normative Aging Study, 1999–2009. Depending on the pollutant, the number of observations ranged from 1,259 to 1,275. Results were adjusted for age, race, height, weight, education level, smoking status, cumulative smoking, season of the medical examination, day of the week, visit number, temperature, relative humidity, asthma, chronic bronchitis, emphysema, methacholine responsiveness, corticosteroids, sympathomimetic α and β, anticholinergics. The IQR (μg/m^3^) was 0.6 for BC, 362 for CO, 15 for NO_2_, 33 for O_3_, 7 for total PM_2.5_,and 5 for nontraffic PM_2.5_.

*Air pollution and lung function*. An interquartile range (IQR) increase in both total and nontraffic PM_2.5_ concentration from the day before lung function measurement (lag 1) to 28-day cumulated exposure was associated with significantly lower FVC by 0.5–2.5% (Figure 1). For BC, only cumulated exposures of 14, 21, and 28 days were significantly associated with FVC, which was 2–5% lower in association with an IQR increase. Significant associations with FEV_1_ were mainly limited to 28-day moving averages for BC, total PM_2.5_ and nontraffic PM_2.5_. As for gases, an IQR increase in CO or NO_2_ concentration from 4- to 28-day moving averages was associated with a significant decrease in FVC by 1–5%; we estimated similar results for FEV_1_, but restricted the analysis to 14- to 28-day moving averages for NO_2_. For all pollutants, estimated effect sizes increased with longer averaging times. BC and NO_2_ had the largest estimated effects on lung function parameters. Associations with IQR increases in 28-day moving average exposures were significantly stronger in participants with emphysema (for BC, PM_2.5_, and NO_2_ with FVC, and for O_3_ and FEV_1_) and in participants with chronic bronchitis (for O_3_ and nontraffic PM_2.5_ with both outcomes, and for PM_2.5_ with FEV_1_) (see Supplemental Material, Table S2).

Further adjusting for cardiovascular diseases (40% of participants), diabetes (17%), and hypertension (82%) did not change the results (not shown). When participants with asthma, emphysema, chronic bronchitis, or methacholine responsiveness (or missing) were excluded (*n* = 254), model estimates were generally consistent with the main analysis, although *p*-values were larger. O_3_ exposure over lag 1 and 3- to 5-day moving averages was associated with significantly lower FVC and FEV_1_. With further controlling for potential survival bias using inverse probability weighting, results varied slightly (see Supplemental Material, Figure S1). Associations of BC, CO, and NO_2_ with lung function were stable or stronger. Associations of O_3_, total PM_2.5_, and nontraffic PM_2.5_ with FVC and FEV_1_ were significant for 28-day cumulative exposures only.

*Effect modification by DNA methylation*. Correlations were relatively high for CpG sites in three of four genes with CpG sites measured at two positions (*CRAT*, *IFN-*γ, and *IL6*), and for one of three pairs of CpG sites in *ICAM1* (0.78 *≤ r ≤* 0.81; see Supplemental Material, Table S3). For the three genes with four to five sites evaluated, there was no evidence of strong correlation between CpG sites within the same gene (*r*_S_ *≤* 0.55). With further adjustment for DNA methylation levels as potential mediators, associations between lung function and air pollutants (28-day moving average) stayed in the same direction; *p*-values were slightly larger and sometimes became nonsignificant for some genes such as *OGG1* (data not shown).

**Figure 2:**
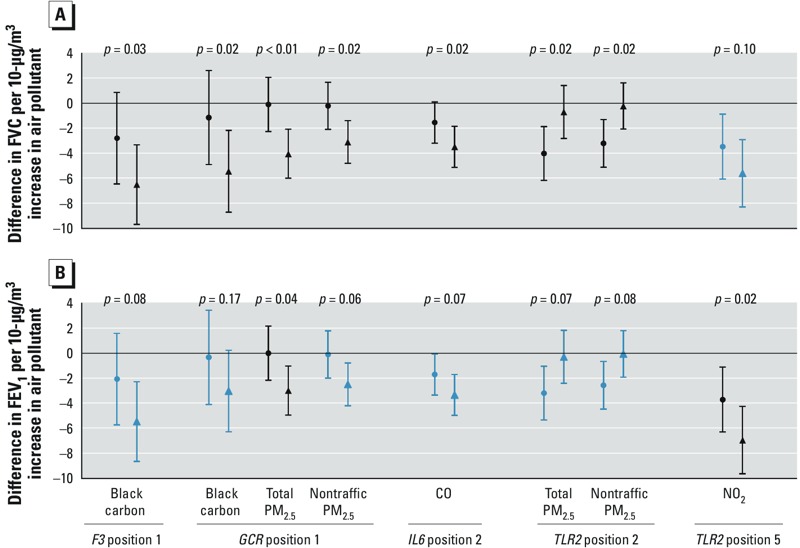
Percent difference in (*A*) FVC and (*B*) FEV_1_ (and 95% CIs) associated with 1 IQR increase in air pollutant concentration (28-day moving average) according to the methylation level [methylation level < median (circles), methylation level ≥ median (triangles)], the Normative Aging Study, 1999–2009. Interactions are in black when statistically significant (*p* < 0.05) and in blue otherwise. Depending on the pollutant and the gene, the number of observations ranged from 767 to 1,208. Results were adjusted for age, race, height, weight, education level, smoking status, cumulative smoking, season of the medical examination, day of the week, visit number, temperature, relative humidity, asthma, chronic bronchitis, emphysema, methacholine responsiveness, corticosteroids, sympathomimetic α and β, anticholinergics, and percent of white blood cells type. The IQR (μg/m^3^) was 0.6 for BC, 362 for CO, 15 for NO_2_, 33 for O_3_, 7 for total PM_2.5_,and 5 for nontraffic PM_2.5_.

Associations between IQR increases in 28-day average air pollutant concentrations and the lung function measures were significantly different between participants with high versus low DNA methylation status at several CpG sites (results for interactions that were significant for at least one outcome are shown in Figure 2; complete results are shown in Supplemental Material, Figure S2).

Associations of BC, total PM_2.5_, and nontraffic PM_2.5_ with FVC were significantly stronger among participants with higher methylation at the CpG site measured in *GCR* (Figure 2), and these interactions remained significant when participants with chronic lung conditions or taking lung-related drugs were excluded from the analysis (data not shown). Associations also were significantly stronger among those with higher methylation at one of five CpG sites measured in *F3* (for BC and FVC) and for those with higher methylation at one of two CpG sites measured in *IL6* (for CO and FVC). Associations of total and nontraffic PM_2.5_ with FVC were significantly stronger among participants with lower methylation at one of the five CpG sites in *TLR2* (position 2), whereas the association between NO_2_ and FEV_1_ was significantly stronger among participants with higher CpG methylation at position 5 in *TLR2*. We did not observe any statistically significant modification of air pollution effects by LINE-1 or Alu methylation (results not shown).

## Discussion

In the elderly population of males examined in the present study, acute exposure to air pollutants (4 hr, lag 0, lag 1) was generally not significantly associated with lung function, but subchronic exposures to all tested pollutants from 3- to 28-day moving averages were significantly associated with lower FVC and FEV_1_ (1–5% lower per IQR increase in air pollution concentrations). Associations with 28-day moving average exposures were stronger in participants with lower methylation levels in one of five CpG sites evaluated in *TLR2* (position 2), and stronger among participants with higher methylation in *GCR* (one CpG site evaluated), *TLR2* (position 5), *F3* (position 1), and *IL6* (position 2). To our knowledge, this is the first study to report associations of short- to medium-term air pollutant exposures with FVC and FEV_1_ in an elderly population, and the first to report evidence of epigene–environment interactions on lung function measures.

A study performed in the elderly reported a significant negative association between the peak expiratory flow and PM_2.5_ and PM_10_ on the same day and up to 4 lagged days (Lee et al. 2007). Epidemiological studies have produced heterogeneous results regarding the lag structure, and very few have explored exposure windows up to 1 month. In adults and schoolchildren, lower FVC and FEV_1_ were associated with increases in CO, NO_2_, PM_10_, and O_3_ from the examination day up to 3 days before (Chang et al. 2012; Hoek et al. 1993; Moshammer et al. 2006; Schindler et al. 2001). In contrast, other studies in adults have reported no associations of current and previous-day exposures to NO_2_, O_3_, or particles with FEV_1_ or FVC (Peacock et al. 2011; Steinvil et al. 2009; Trenga et al. 2006), and/or delayed associations within 3–7 days (Barraza-Villarreal et al. 2008; de Hartog et al. 2009; Hoek and Brunekreef 1993; Steinvil et al. 2009). In terms of effect estimates, Schindler et al. (2001) reported a decrease of 0.4–0.8% in FVC and FEV_1_ associated with a 10-μg/m^3^ increase in the 4-day cumulated exposure to NO_2_ in never-smoker adults, which is consistent with the 1.1% decrease in FVC we estimated in association with a 15-μg/m^3^ increase in the 4-day NO_2_ moving average. However, such comparisons are usually made difficult by different modeling choices across studies.

Our results suggested mainly adverse effects of traffic pollutants represented by BC, CO, and NO_2_ on lung function, whereas results for nontraffic PM_2.5_ and O_3_ were less clear. In Boston, BC is a marker of traffic particles influenced by both local traffic, with a morning peak, and long-range transported traffic particles (Park et al. 2007). NO_2_, a lower-airway irritant, is mainly a secondary pollutant, which in Boston mostly originates from traffic and regional sources including power plants and vehicular emissions (Rattigan et al. 2010). There is limited physiologic rationale for an association of CO with reduced lung function. In fact, CO is used therapeutically for acute respiratory distress syndrome. Rather, we interpret its association as an indication of traffic co-pollutants such as ultrafine particles, BC, or NO_2_, for which CO could serve as a surrogate. In contrast, controlled exposure to diesel exhaust, rich in BC and NO_x_ (nitrogen oxides), has been shown to produce pulmonary inflammation (Salvi et al. 1999). Associations with nontraffic PM_2.5_ were attenuated when adjusted for potential survival bias, suggesting that nontraffic PM_2.5_ was not or was only slightly associated with lung function. Although some studies have reported evidence of short-term effects of O_3_ on lung function (Chang et al. 2012), others have failed to demonstrate an association (Barraza-Villarreal et al. 2008). A previous analysis in the Normative Aging Study covering the period 1995–2005 estimated significantly lower FEV_1_ and FVC in association with O_3_ increases (Alexeeff et al. 2007). The present analysis with longer follow-up was able to reproduce those results only when participants with chronic lung diseases were excluded. We do not have any biological explanation for this result, but it is in agreement with the results reported by Lagorio et al. (2006), who did not find any effect of O_3_ on lung function in adults with preexisting conditions such as asthma and COPD.

There is a growing body of evidence showing that innate immunity response, and particularly TLRs, are implied in pulmonary inflammation (Poole et al. 2011). TLRs recognize damage-associated molecular patterns and activate nuclear factor kappa–light-chain-enhancer of activated B cells (NF-κB), which initiate the production of numerous cytokines and host-defense molecules. *TLR2* is expressed primarily in blood leukocytes and lung (Zarember and Godowski 2002). Consistent with this, our previous results showed that lower methylation in *TLR2* was associated with decreased lung function (Lepeule et al. 2012). *TLRs* may also be involved in the development of respiratory diseases (Smit et al. 2009) and in gene–environment interactions. For instance, a placebo-controlled intervention study conducted in 916 children from the Netherlands reported that *TLR2* variants influenced the susceptibility of developing asthma in response to NO_2_ and PM_2.5_ exposure (Kerkhof et al. 2010). By showing that effects of NO_2_, total PM_2.5_, and nontraffic PM_2.5_ on lung function varied significantly according to the methylation status in the promoter region of *TLR2,* our study provides preliminary support for epigenetic modification of susceptibility to effects of air pollution on lung function. We found inconsistent results between position 2 and position 5 for *TLR2.* This may be interpreted as evidence against a causal effect of *TLR*2 on susceptibility. Also, Herman and Baylin (2003) have reported that hypomethylated regions in gene promoters are flanked to either side by methylated cytosines. Position 5 in the *TLR2* sequence analyzed may represent a neighboring methylation site not directly involved in *TLR2* gene suppression, yet hypermethylated in active genes.

*GCR* is an antiinflammatory gene strongly related to stress (Miller et al. 2008), inflammation (Smoak and Cidlowski 2004), and lung diseases, which encodes a glucocorticoid protein receptor expressed in the lungs (AceView 2013). The main ligands of GCR are corticosteroids. Upon ligand binding, the activated GCR can switch on antiinflammatory genes and switch off inflammatory genes that encode signaling molecules and inflammatory receptors, which are regulated by proinflammatory transcription factors such as NF-κB cells and activator protein. Poor glucocorticoid responses related to genetic polymorphisms in *GCR* have been suggested to increase COPD risk and severity (Schwabe et al. 2009). Our results indicated a stronger association between particulate air pollution (BC, PM_2.5_, nontraffic PM_2.5_) and reduced lung function in participants with higher methylation at a CpG site in the *GCR* promoter region.

*F3* encodes coagulation factor III (i.e., tissue factor), a major player in hemostasis. Previous results suggest that the promoter sequence we studied could regulate the gene (Mackman et al. 2007). We found stronger associations between BC and FVC among individuals with higher methylation at one of five CpG sites measured in *F3*. Similarly, we found stronger associations between CO and FVC among men with higher methylation at one of two CpG sites evaluated in *IL6. IL6* encodes a protein that may act as both pro- and antiinflammatory cytokine and previous results in our cohort did not find any association of *IL6* with lung function (Litonjua et al. 2003).

Altogether, these results suggest that methylation in inflammation- and immunity-related genes might contribute to effect of air pollution. DNA methylation marks are established mainly during early life, and although thought to be a fairly stable measure, DNA methylation has been associated with aging (Madrigano et al. 2012). In our data, almost all associations between air pollutants and lung function remained after adjustment for DNA methylation, which suggests that pulmonary effects of air pollutants were not mediated by methylation. We checked the locations at which we measured DNA methylation on UCSC Genome Build hg19 (http://genome.ucsc.edu/cgi-bin/hgGateway). We specifically designed the assays to exclude SNPs (single-nucleotide polymorphisms) from the CpG sites analyzed and the sequences hybridized by the polymerase chain reaction and pyrosequencing primers. However, because dense genotyping data are not available for this study, we cannot exclude effects from SNPs nearby or remotely located relative to the sequence analyzed. We generally observed stronger associations of air pollutants with FVC than with FEV_1_, suggesting that air pollution may affect more of the smaller airways and therefore is associated with restrictive lung diseases. In the elderly, particle clearance might be less efficient or impaired by other dysfunctions. The apparently small effects of air pollution on lung function should not be underestimated because they might be well tolerated by healthy population, but become life threatening for elderly or ill subjects (Gouveia and Fletcher 2000). In addition, repeated occurrences of short-term decrements in lung function and accompanying inflammation may play some role in the development of long-term decrements.

We acknowledge several limitations of our study. First, as often in air pollution studies, we did not account for indoor exposure to air pollutants. Assuming that the elderly have reduced outdoor activities, indoor sources might be a larger contributor to personal exposure than in other populations. Under the assumption that indoor and outdoor particles are identical, errors due to indoor exposures have been shown of Berkson type, which is expected to increase standard errors (Zeger et al. 2000). If this assumption of identical indoor and outdoor particles does not hold, the estimated effect would generally be biased downward. Another source of measurement error lies in the low spatial resolution of our exposure model, limited to a few monitoring stations for the entire study area. However, short-term effects studies tend to focus on temporal variation rather than spatial variation. Assuming that daily variation in air pollutant concentrations is homogeneous across the study area might have introduced random noise in exposure estimates, which would tend to underestimate the association with lung function. Therefore it is unlikely that any exposure measurement error would bias the effect away from the null, and this might explain the lack of statistical significance of our PM_2.5_-related results. Second, study results were observed in a cohort of elderly men, which may limit the generalizability of our results to similar populations. Third, for the sake of feasibility, we dichotomized DNA methylation at each CpG site to examine interactions with pollutants concentration. Interactions were analyzed for multiple CpG sites, pollutants, and outcomes, and require caution regarding potential false-positive findings. Finally, although we adjusted for the percentage of white blood cell types, we did not have data available for lymphocyte subsets. Therefore, we cannot rule out the possibility that the interactions are with those subset prevalences and not with the methylation per se.

## Conclusion

This study adds to the growing body of literature on short- and long-term effects of traffic-related air pollutants mainly studied in children and young adults, by showing that subchronic but not acute exposure is associated with lower lung function in the elderly. As for the mechanisms, alongside previous studies on genetic variation in inflammatory and immunity genes and respiratory impairment susceptibility, our results suggest that epigenetic mechanisms related to inflammation and immunity may also be implicated in these associations.

## Supplemental Material

(666 KB) PDF

## References

[r1] AceView. (2013). The AceView genes.. http://www.ncbi.nlm.nih.gov/IEB/Research/Acembly/.

[r2] Alexeeff SE Litonjua AA Sparrow D Vokonas PS Schwartz J 2007 Statin use reduces decline in lung function: VA Normative Aging Study. Am J Respir Crit Care Med 176 742 747; 10.1164/rccm.200705-656OC 17673694 PMC2020828

[r3] Baccarelli A Wright R Bollati V Litonjua A Zanobetti A Tarantini L 2010 Ischemic heart disease and stroke in relation to blood DNA methylation. Epidemiology 21 819 828; 10.1097/EDE.0b013e3181f20457 20805753 PMC3690659

[r4] Barraza-Villarreal A Sunyer J Hernandez-Cadena L Escamilla-Nunez MC Sienra-Monge JJ Ramirez-Aguilar M 2008 Air pollution, airway inflammation, and lung function in a cohort study of Mexico City schoolchildren. Environ Health Perspect 116 832 838; 10.1289/ehp.10926 18560490 PMC2430242

[r5] Bell B, Rose CL, Damon A (1966). The Veterans Administration longitudinal study of healthy aging.. Gerontologist.

[r6] Brunekreef B, Dockery DW, Krzyzanowski M (1995). Epidemiologic studies on short-term effects of low levels of major ambient air pollution components.. Environ Health Perspect.

[r7] Budulac SE Boezen HM Hiemstra PS Lapperre TS Vonk JM Timens W 2012 Toll-like receptor (TLR2 and TLR4) polymorphisms and chronic obstructive pulmonary disease. PLoS ONE 7 e43124; 10.1371/journal.pone.0043124 22952638 PMC3429472

[r8] Chang YK Wu CC Lee LT Lin RS Yu YH Chen YC 2012 The short-term effects of air pollution on adolescent lung function in Taiwan. Chemosphere 87 26 30; 10.1016/j.chemosphere.2011.11.048 22189374

[r9] Chatham M, Bleecker ER, Norman P, Smith PL, Mason P (1982). A screening test for airways reactivity. An abbreviated methacholine inhalation challenge.. Chest.

[r10] de Hartog JJ Ayres JG Karakatsani A Analitis A Brink HT Hameri K 2009 Lung function and indicators of exposure to indoor and outdoor particulate matter among asthma and COPD patients. Occup Environ Med 67 2 10; 10.1136/oem.2008.040857 19736175

[r11] Ferris BG (1978). Epidemiology Standardization Project (American Thoracic Society).. Am Rev Respir Dis.

[r12] Gouveia N Fletcher T 2000 Time series analysis of air pollution and mortality: effects by cause, age and socioeconomic status. J Epidemiol Community Health 54 750 755; 10.1136/jech.54.10.750 10990478 PMC1731551

[r13] Hashimshony T Zhang J Keshet I Bustin M Cedar H 2003 The role of DNA methylation in setting up chromatin structure during development. Nat Genet 34 187 192; 10.1038/ng1158 12740577

[r14] He JQ Foreman MG Shumansky K Zhang X Akhabir L Sin DD 2009 Associations of IL6 polymorphisms with lung function decline and COPD. Thorax 64 698 704; 10.1136/thx.2008.111278 19359268 PMC2859187

[r15] Herman JG Baylin SB 2003 Gene silencing in cancer in association with promoter hypermethylation. N Engl J Med 349 2042 2054; 10.1056/NEJMra023075 14627790

[r16] Hernan MA, Lanoy E, Costagliola D, Robins JM (2006). Comparison of dynamic treatment regimes via inverse probability weighting.. Basic Clin Pharmacol Toxicol.

[r17] Hoek G, Brunekreef B (1993). Acute effects of a winter air pollution episode on pulmonary function and respiratory symptoms of children.. Arch Environ Health.

[r18] Hoek G, Fischer P, Brunekreef B, Lebret E, Hofschreuder P, Mennen MG (1993). Acute effects of ambient ozone on pulmonary function of children in the Netherlands.. Am Rev Respir Dis.

[r19] Islam T Breton C Salam MT McConnell R Wenten M Gauderman WJ 2009 Role of inducible nitric oxide synthase in asthma risk and lung function growth during adolescence. Thorax 65 139 145; 10.1136/thx.2009.114355 19996333

[r20] Kerkhof M Postma DS Brunekreef B Reijmerink NE Wijga AH de Jongste JC 2010 Toll-like receptor 2 and 4 genes influence susceptibility to adverse effects of traffic-related air pollution on childhood asthma. Thorax 65 690 697; 10.1136/thx.2009.119636 20685742

[r21] Lagorio S Forastiere F Pistelli R Iavarone I Michelozzi P Fano V 2006 Air pollution and lung function among susceptible adult subjects: a panel study. Environ Health 5 11; 10.1186/1476-069X-5-11 16674831 PMC1475828

[r22] Lange NE Sordillo J Tarantini L Bollati V Sparrow D Vokonas P 2012 Alu and LINE-1 methylation and lung function in the Normative Aging Study. BMJ Open 2 5 e001231; 10.1136/bmjopen-2012-001231 PMC3488751 23075571

[r23] Lee JT Son JY Cho YS 2007 The adverse effects of fine particle air pollution on respiratory function in the elderly. Sci Total Environ 385 28 36; 10.1016/j.scitotenv.2007.07.005 17692897

[r24] Lepeule J, Baccarelli A, Tarantini L, Motta V, Cantone L, Litonjua A (2012). Gene promoter methylation is associated with lung function in the elderly.. Epigenetics.

[r25] Litonjua AA Sparrow D Guevarra L O’Connor GT Weiss ST Tollerud DJ 2003 Serum interferon-gamma is associated with longitudinal decline in lung function among asthmatic patients: the Normative Aging Study. Ann Allergy Asthma Immunol 90 422 428; 10.1016/S1081-1206(10)61827-3 12722965

[r26] Mackman N Tilley RE Key NS 2007 Role of the extrinsic pathway of blood coagulation in hemostasis and thrombosis. Arterioscler Thromb Vasc Biol 27 1687 1693; 10.1161/ATVBAHA.107.141911 17556654

[r27] Madrigano J Baccarelli A Mittleman MA Sparrow D Vokonas PS Tarantini L 2012 Aging and epigenetics: longitudinal changes in gene-specific DNA methylation. Epigenetics 7 63 70; 10.4161/epi.7.1.18749 22207354 PMC3329504

[r28] Miller GE Chen E Sze J Marin T Arevalo JM Doll R 2008 A functional genomic fingerprint of chronic stress in humans: blunted glucocorticoid and increased NF-κB signaling. Biol Psychiatry 64 266 272; 10.1016/j.biopsych.2008.03.017 18440494 PMC2581622

[r29] Moshammer H Hutter HP Hauck H Neuberger M 2006 Low levels of air pollution induce changes of lung function in a panel of schoolchildren. Eur Respir J 27 1138 1143; 10.1183/09031936.06.00089605 16455832

[r30] Park SK, O’Neill MS, Stunder BJ, Vokonas PS, Sparrow D, Koutrakis P (2007). Source location of air pollution and cardiac autonomic function: trajectory cluster analysis for exposure assessment.. J Expo Sci Environ Epidemiol.

[r31] Peacock JL Anderson HR Bremner SA Marston L Seemungal TA Strachan DP 2011 Outdoor air pollution and respiratory health in patients with COPD. Thorax 66 591 596; 10.1136/thx.2010.155358 21459856

[r32] Poole JA Wyatt TA Kielian T Oldenburg P Gleason AM Bauer A 2011 Toll-like receptor 2 (TLR2) regulates organic dust-induced airway inflammation. Am J Respir Cell Mol Biol 45 711 719; 10.1165/rcmb.2010-0427OC 21278324 PMC3208620

[r33] Rattigan OV, Felton HD, Bae MS, Schwab JJ, Demerjian KL (2010). Multi-year hourly PM_2.5_ carbon measurements in New York: diurnal, day of week and seasonal patterns.. Atmos Environ.

[r34] Salvi S, Blomberg A, Rudell B, Kelly F, Sandstrom T, Holgate ST (1999). Acute inflammatory responses in the airways and peripheral blood after short-term exposure to diesel exhaust in healthy human volunteers.. Am J Respir Crit Care Med.

[r35] Sandstrom T, Frew AJ, Svartengren M, Viegi G (2003). The need for a focus on air pollution research in the elderly.. Eur Respir J Suppl.

[r36] Schindler C, Kunzli N, Bongard JP, Leuenberger P, Karrer W, Rapp R (2001). Short-term variation in air pollution and in average lung function among never-smokers. The Swiss Study on Air Pollution and Lung Diseases in Adults (SAPALDIA).. Am J Respir Crit Care Med.

[r37] Schwabe K, Vacca G, Duck R, Gillissen A (2009). Glucocorticoid receptor gene polymorphisms and potential association to chronic obstructive pulmonary disease susceptibility and severity.. Eur J Med Res.

[r38] Schwartz J Litonjua A Suh H Verrier M Zanobetti A Syring M 2005 Traffic related pollution and heart rate variability in a panel of elderly subjects. Thorax 60 455 461; 10.1136/thx.2004.024836 15923244 PMC1747419

[r39] Sin DD Man SF 2008 Interleukin-6: a red herring or a real catch in COPD? Chest 133 4 6; 10.1378/chest.07-2085 18187736

[r40] Smit LA Siroux V Bouzigon E Oryszczyn MP Lathrop M Demenais F 2009 *CD14* and toll-like receptor gene polymorphisms, country living, and asthma in adults. Am J Respir Crit Care Med 179 363 368; 10.1164/rccm.200810-1533OC 19096003

[r41] Smoak KA, Cidlowski JA (2004). Mechanisms of glucocorticoid receptor signaling during inflammation.. Mech Ageing Dev.

[r42] Sparrow D, O’Connor G, Colton T, Barry CL, Weiss ST (1987). The relationship of nonspecific bronchial responsiveness to the occurrence of respiratory symptoms and decreased levels of pulmonary function. The Normative Aging Study.. Am Rev Respir Dis.

[r43] Steinvil A Fireman E Kordova-Biezuner L Cohen M Shapira I Berliner S 2009 Environmental air pollution has decremental effects on pulmonary function test parameters up to one week after exposure. Am J Med Sci 338 273 279; 10.1097/MAJ.0b013e3181adb3ed 19726973

[r44] Stenvinkel P Karimi M Johansson S Axelsson J Suliman M Lindholm B 2007 Impact of inflammation on epigenetic DNA methylation—a novel risk factor for cardiovascular disease? J Intern Med 261 488 499; 10.1111/j.1365-2796.2007.01777.x 17444888

[r45] Sunyer J Pistelli R Plana E Andreani M Baldari F Kolz M 2008 Systemic inflammation, genetic susceptibility and lung function. Eur Respir J 32 92 97; 10.1183/09031936.00052507 18385179

[r46] Tost J, Gut IG (2007). DNA methylation analysis by pyrosequencing.. Nat Protoc.

[r47] Trenga CA Sullivan JH Schildcrout JS Shepherd KP Shapiro GG Liu LJ 2006 Effect of particulate air pollution on lung function in adult and pediatric subjects in a Seattle panel study. Chest 129 1614 1622; 10.1378/chest.129.6.1614 16778283

[r48] U.S. Department of Health and Human Services. (2014). Aging Statistics.. http://www.aoa.gov/aoaroot/aging_statistics/index.aspx.

[r49] Yang IA Fong KM Zimmerman PV Holgate ST Holloway JW 2008 Genetic susceptibility to the respiratory effects of air pollution. Thorax 63 555 563; 10.1136/thx.2007.079426 18511640

[r50] Zanobetti A Schwartz J 2007 Particulate air pollution, progression, and survival after myocardial infarction. Environ Health Perspect 115 769 775; 10.1289/ehp.9201 17520066 PMC1867961

[r51] Zarember KA, Godowski PJ (2002). Tissue expression of human Toll-like receptors and differential regulation of Toll-like receptor mRNAs in leukocytes in response to microbes, their products, and cytokines.. J Immunol.

[r52] Zeger SL, Thomas D, Dominici F, Samet JM, Schwartz J, Dockery D (2000). Exposure measurement error in time-series studies of air pollution: concepts and consequences.. Environ Health Perspect.

