# Supplementary material for: Epigenetic Influences on Associations between Air Pollutants and Lung Function in Elderly Men: The Normative Aging Study
Source: Environ Health Perspect. 2014 Mar 6;122(6):566–72. doi: 10.1289/ehp.1206458 (PMC4050500; doi:10.1289/ehp.1206458)
Supplement: (666 KB) PDF [file ehp.1206458.s001.pdf]

Supplemental Material

Epigenetic Influences on Associations between Air Pollutants and Lung Function in Elderly Men: The Normative Aging Study

Johanna Lepeule, Marie-Abele Catherine Bind, Andrea A. Baccarelli, Petros Koutrakis, Letizia Tarantini, Augusto Litonjua, David Sparrow, Pantel Vokonas, and Joel D. Schwartz

Table of contents **Page**

Figure S1. Sensitivity analysis adjusting for potential survival bias using inverse probability weighting: percent difference in forced vital capacity (FVC, black circles) and forced expiratory volume in 1 second (FEV_1, grey circles) (and 95% confidence intervals (bars)) associated with one interquartile range increase in air pollutant concentration in 776 men participating to the Normative Aging Study, 1999-2009. **3**

Figure S2. Percent difference in forced vital capacity (FVC) (in black) and forced expiratory volume in 1 second (FEV_1) (in grey) (and 95% confidence intervals (bars)) associated with one interquartile range increase in air pollutant concentration (28-days moving average) according to the methylation level (methylation level<median (circles), methylation level ≥ median (triangles)), the Normative Aging Study, Boston metropolitan area, 1999-2009 **4**

Table S1. Localization of gene promoters and regions amplified **7**

Table S2. Percent difference in forced vital capacity (FVC) and forced expiratory volume in 1 second (FEV_1) associated with one interquartile range increase in air pollutant concentration (28-days moving average) according to the presence of chronic lung conditions, in 776 men who underwent 1515 visits, the Normative Aging Study, 1999-2009 **8**

Table S3. Spearman correlation coefficients between CpG sites within the same gene in 776 men who underwent 1515 visits, the Normative Aging Study, Boston metropolitan area, 1999-2009 **9**

Figure S1. Sensitivity analysis adjusting for potential survival bias using inverse probability weighting: percent difference in forced vital capacity (FVC, black circles) and forced expiratory volume in 1 second (FEV_1, grey circles) (and 95% confidence intervals (bars)) associated with one interquartile range increase in air pollutant concentration in 776 men participating to the Normative Aging Study, 1999-2009.

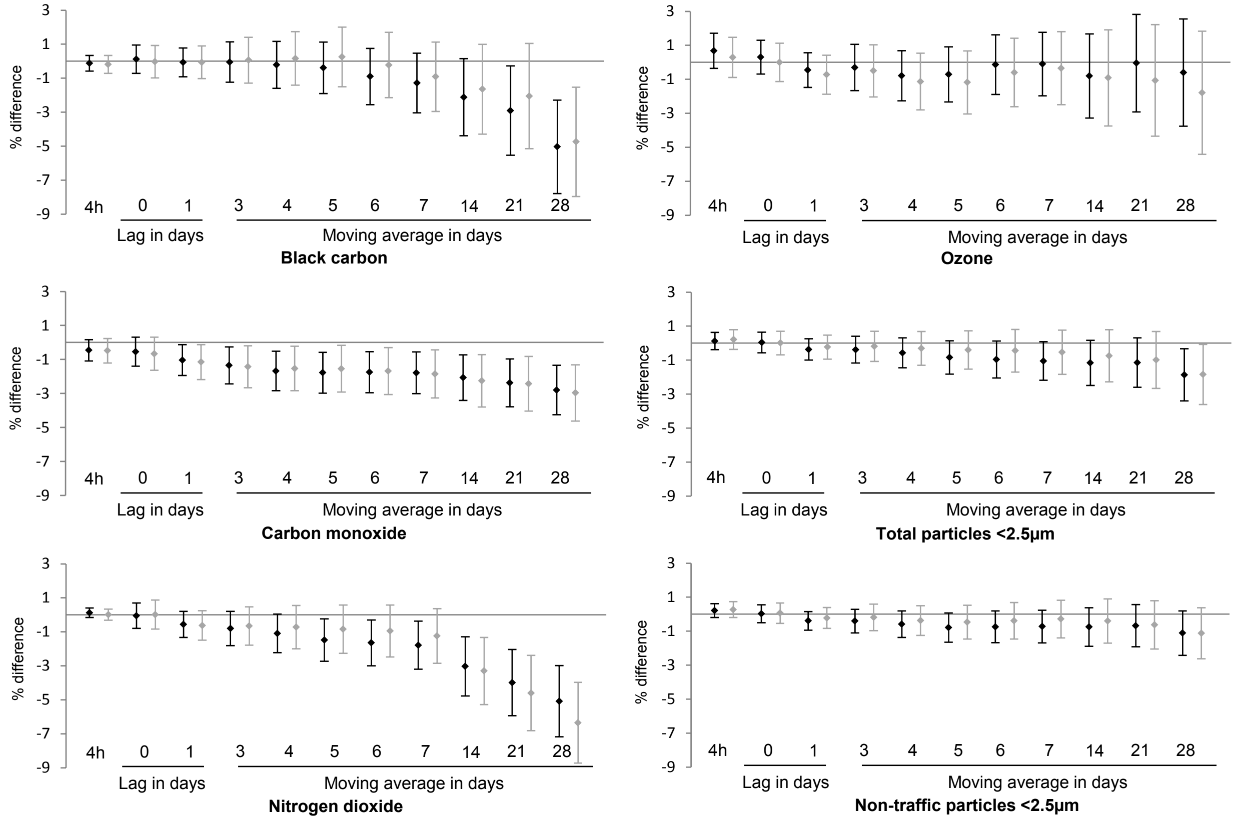

Depending on the pollutant, the number of observations ranged from 1251 to 1271. Results are adjusted for age, race, height, weight, education level, smoking status, cumulative smoking, season of the medical exam, day of the week, visit number, temperature, relative humidity, asthma, chronic bronchitis, emphysema, methacholine responsiveness, corticosteroids, sympathomimetic alpha and beta, anticholinergics. The interquartile range ($\mu g/m^3$) was 0.6 for black carbon, 360 for carbon monoxide, 15 for nitrogen dioxide, 33 for ozone, 7 for particles < 2.5 µm and 4 for non-traffic particles < 2.5 µm.

Figure S2. Percent difference in forced vital capacity (FVC) (in black) and forced expiratory volume in 1 second (FEV_1) (in grey) (and 95% confidence intervals (bars)) associated with one interquartile range increase in air pollutant concentration (28-days moving average) according to the methylation level (methylation level < median (circles), methylation level ≥ median (triangles)), the Normative Aging Study, Boston metropolitan area, 1999-2009.

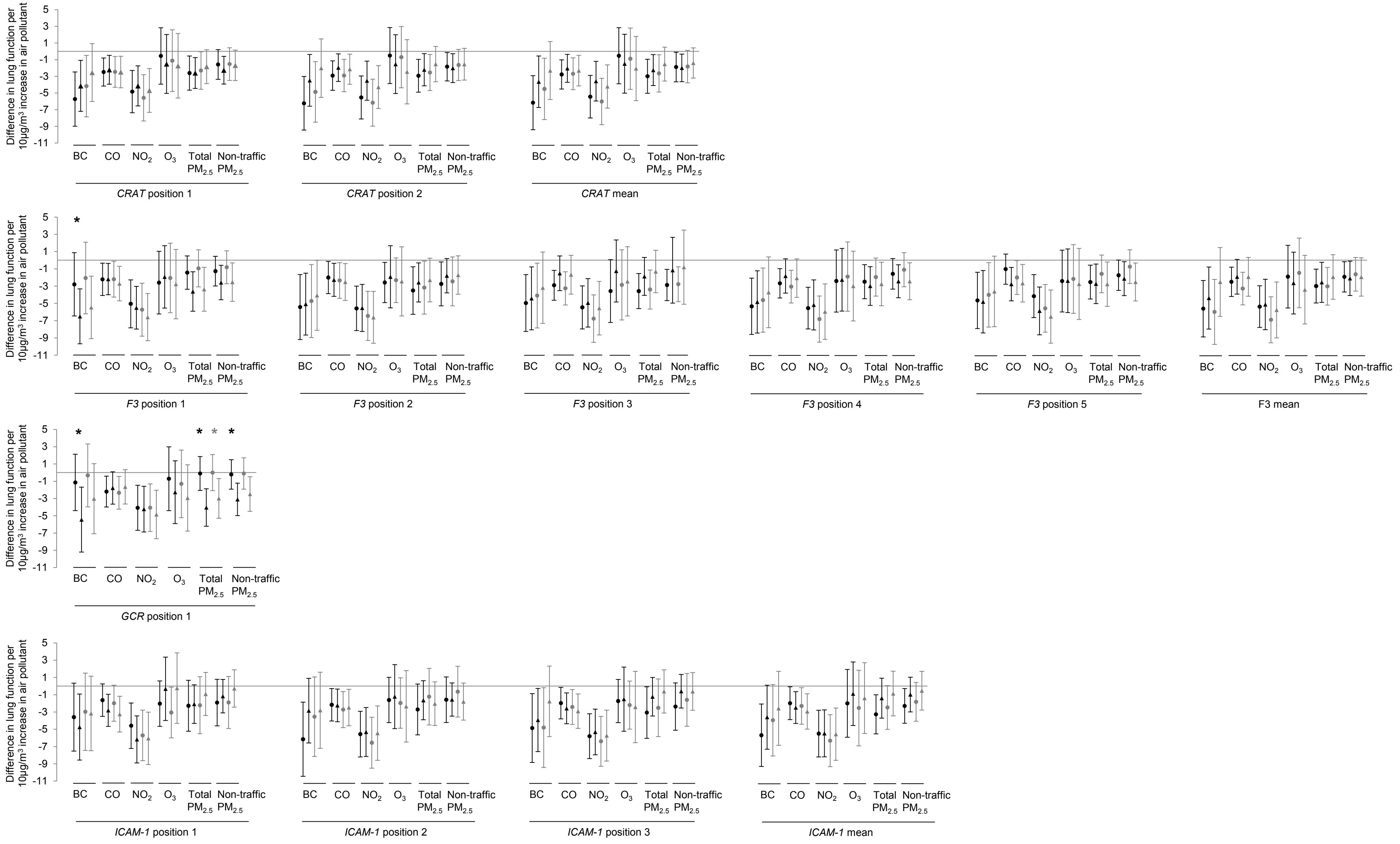

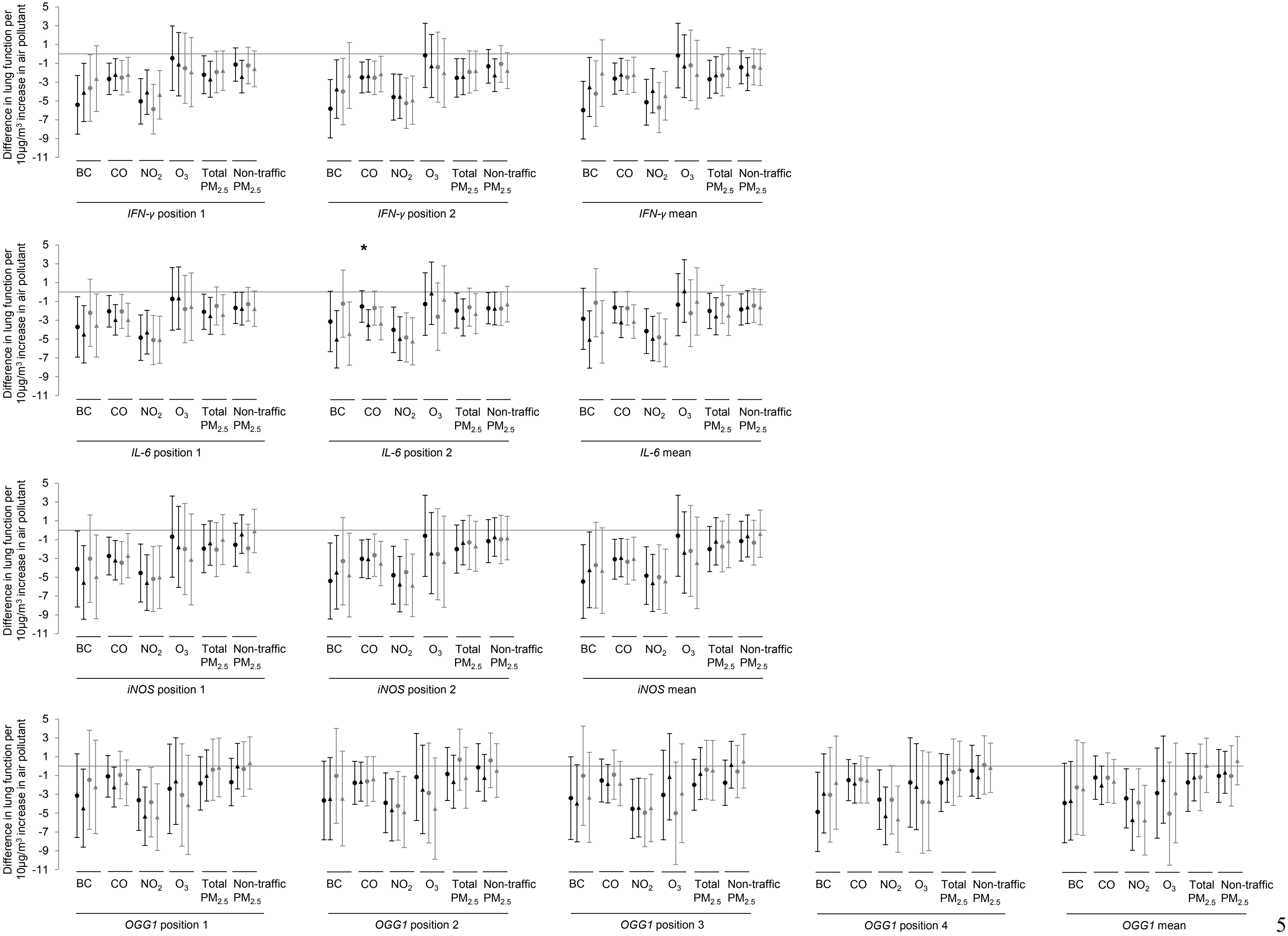
Difference in lung function per 10μg/m³ increase in air pollutant
BC
CO
NO2
O3
Total PM2.5
Non-traffic PM2.5
IFN-γ position 1
IFN-γ position 2
IFN-γ mean
IL-6 position 1
IL-6 position 2
IL-6 mean
iNOS position 1
iNOS position 2
iNOS mean
OGG1 position 1
OGG1 position 2
OGG1 position 3
OGG1 position 4
OGG1 mean

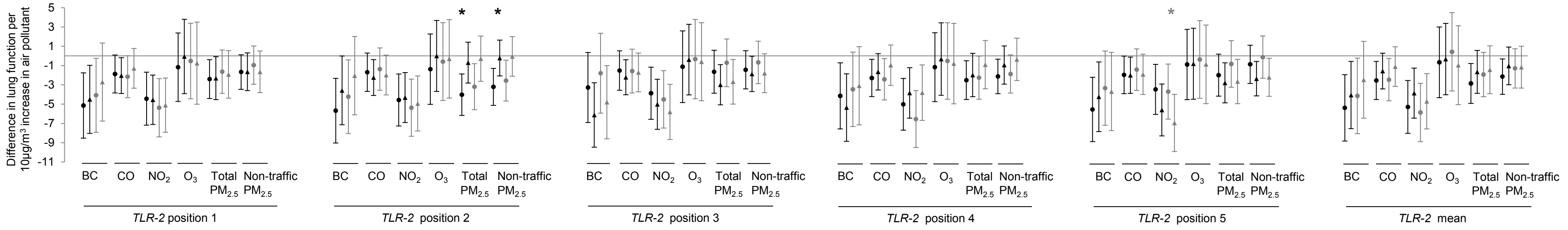

*Significant interactions ($p < 0.05$).

CRAT = Carnitine O-acetyltransferase, F3 =coagulation factor-3, GCR =glucocorticoid receptor, ICAM-1, intercellular adhesion molecule, IFN-*γ, interferon-gamma, IL-6 =interleukin-6, iNOS,* inducible nitric oxide synthase, OGG1, *8-oxoguanine DNA glycosylase 1, TLR-2, toll-like receptor-2.* Results were adjusted for age, race, height, weight, education level, smoking status, cumulative smoking, season of the medical exam, day of the week, visit number, temperature, relative humidity, asthma, chronic bronchitis, emphysema, methacholine responsiveness, corticosteroids, sympathomimetic alpha and beta, anticholinergics, and % of white blood cells type. The interquartile range ($\mu g/m^3$) was 0.6 for black carbon, 362 for carbon monoxide (CO), 15 for nitrogen dioxide (NO_2), 33 for ozone (O_3), 7 for total particles < 2.5 µm ($PM_{2.5}$) and 5 for non-traffic $PM_{2.5}$.

Table S1. Localization of gene promoters and regions amplified.

Gene	Chromosome	Promoter *Start-end*	Amplicon *Start-end*	Location	Promoter	CpGs (position)
CRAT	9	130912702-130913404	130912776-130912862	CpG island	yes	130912824 (pos1)<br>130912806 (pos2)
F3	1	94779671-94780502	94779878-94780068	CpG island	yes	94779947 (pos1)<br>94779950 (pos2)<br>94779956 (pos3)<br>94779958 (pos4)<br>94779974 (pos5)
GCR	5	142760496-142761097	142760531-142760806	non-CpG island	yes	142760565
ICAM-1	19	10242017-10242937	10242034-10242283	CpG island	yes	10242236 (pos1)<br>10242225 (pos2)<br>10242218 (pos3)
IFN-γ	12	66839561-66840293	66840120-66840260	non-CpG island		66840192 (pos1)<br>66840186 (pos2)
IL-6	7	22732791-22733685	22733758-22733893	non-CpG island	no	22733847 (pos1)<br>22733841 (pos2)
iNOS	17	23149861-23150461	23149873-23149990	non-CpG island	yes	23149929 (pos1)<br>23149936 (pos2)
OGG1	3	9766128-9766775	9766288-9766514	CpG island	yes	9766356 (pos1)<br>9766366 (pos2)<br>9766373 (pos3)<br>9766380 (pos4)
TLR-2	4	154824391-154824991	154824566-154824754	CpG island	yes	154824709 (pos1)<br>154824713 (pos2)<br>154824715 (pos3)<br>154824723 (pos4)<br>154824727 (pos5)

Table S2. Percent difference in forced vital capacity (FVC) and forced expiratory volume in 1 second (FEV_1) associated with one interquartile range increase in air pollutant concentration (28-days moving average) according to the presence of chronic lung conditions, in 776 men who underwent 1515 visits, the Normative Aging Study, 1999-2009.

**Pollutant**	**Chronic lung condition***	**n**	**FVC: β (95%CI)**	**FVC: p-value interaction**[a]	**FEV_1: β (95%CI)**	**FEV_1: p-value interaction**[a]
Black carbon	No emphysema	1241	-4.9 (-7.7, -2.0)	0.006		
	Emphysema	34	-17.2 (-24.2, -8.7)			
NO_2	No emphysema	1241	-4.4 (-6.4, -2.4)	0.04		
	Emphysema	34	-12.9 (-21.1, -4.6)			
O_3	No emphysema	1241			-1.7 (-5.0, 1.6)	0.03
	Emphysema	34			7.4 (-1.3, 16.1)	
O_3	No chronic bronchitis	1204	-0.4 (-3.4, 2.6)	0.03	-1.0 (-4.3, 2.2)	0.04
	Chronic bronchitis	71	-5.9 (-11.6, -0.2)		-6.8 (-13.0, -0.6)	
$PM_{2.5}$	No emphysema	1241	-2.9 (-4.5, -1.3)	0.01		
	Emphysema	34	-15.3 (-24.8, -5.6)			
$PM_{2.5}$	No chronic bronchitis	1204			-2.0 (-3.8, -0.2)	0.02
	Chronic bronchitis	71			-7.4 (-11.8, -3.0)	
$PM_{2.5}$ non-traffic	No chronic bronchitis	1204	-1.8 (-3.1, -0.4)	0.04	-1.3 (-2.8, 0.2)	0.009
	Chronic bronchitis	71	-5.7 (-9.4, -2.0)		-6.8 (-10.7, -2.8)	

Due to some missing values, the number of observations examined was 1275.

Results were adjusted for age, race, height, weight, education level, smoking status, cumulative smoking, season of the medical exam, day of the week, visit number, temperature, relative humidity, asthma, chronic bronchitis, emphysema, methacholine responsiveness, corticosteroids, sympathomimetic alpha and beta, anticholinergics.

The interquartile range ($\mu g/m^3$) was 0.6 for black carbon, 15 for nitrogen dioxide (NO_2), 33 for ozone (O_3), 7 for total particles < 2.5 µm ($PM_{2.5}$) and 5 for non-traffic $PM_{2.5}$.

*Chronic lung conditions considered were emphysema, chronic bronchitis, methacholine responsiveness (as an objective indicator of asthma) and COPD (defined as GOLD stage II ($FEV_1/FVC < 70\%$ and $FEV_1 < 80\%$ predicted) or higher). Only results pertaining to significant interactions between pollutants and chronic lung conditions are shown.

[a]Interaction between chronic lung conditions and air pollutant concentration.

Table S3. Spearman correlation coefficients between CpG sites within the same gene in 776 men who underwent 1515 visits, the Normative Aging Study, 1999-2009.

	***CRAT* pos1**	***CRAT* pos2**	***F3* pos1**	***F3* pos2**	***F3* pos3**	***F3* pos4**	***F3* pos5**	***ICAM-1* pos1**	***ICAM-1* pos2**	***ICAM-1* pos3**	***IFN-γ* pos1**	***IFN-γ* pos2**	***IL-6* pos1**	***IL-6* pos2**	***iNOS* pos1**	***iNOS* pos2**	***OGG1* pos1**	***OGG1* pos2**	***OGG1* pos3**	***OGG1* pos4**	***TLR-2* pos1**	***TLR-2* pos2**	***TLR-2* pos3**	***TLR-2* pos4**	***TLR-2* pos5**
CRAT																									
pos1	1.00																								
pos2	0.79	1.00																							
mean	0.87	0.99																							
F3																									
pos1			1.00																						
pos2			0.26	1.00																					
pos3			0.29	0.18	1.00																				
pos4			0.29	0.32	0.44	1.00																			
pos5			0.27	0.16	0.46	0.39	1.00																		
mean			0.52	0.44	0.73	0.62	0.72																		
ICAM-1																									
pos1								1.00																	
pos2								0.81	1.00																
pos3								0.55	0.51	1.00															
mean								0.91	0.87	0.79															
IFN-γ																									
pos1											1.00														
pos2											0.78	1.00													
mean											0.96	0.92													
IL-6																									
pos1													1.00												
pos2													0.79	1.00											
mean													0.95	0.94											
iNOS																									
pos1															1.00										
pos2															0.42	1.00									
mean															0.82	0.84									
OGG1																									
pos1																	1.00								
pos2																	0.02	1.00							
pos3																	-0.03	0.02	1.00						
pos4																	0.12	0.14	0.34	1.00					
mean																	0.47	0.55	0.42	0.57					
TLR-2																									
pos1																					1.00				
pos2																					0.44	1.00			
pos3																					0.41	0.38	1.00		
pos4																					0.36	0.31	0.44	1.00	
pos5																					0.27	0.21	0.35	0.42	1.00
mean																					0.68	0.64	0.72	0.72	0.61

Pos = CpG site (see Table S1).

Depending on the gene and position, the sample size ranged from 915 to 1469.
